# Advances in selective activation of muscles for non-invasive motor neuroprostheses

**DOI:** 10.1186/s12984-016-0165-2

**Published:** 2016-06-13

**Authors:** Aikaterini D. Koutsou, Juan C. Moreno, Antonio J. del Ama, Eduardo Rocon, José L. Pons

**Affiliations:** Neural Rehabilitation Group, Cajal Institute, Spanish National Research Council, Madrid, Spain; National Hospital for Spinal Cord Injury, Toledo, Spain; Neural and Cognitive Engineering group, Centro de Automática y Robótica, CAR, Spanish National Research Council, CSIC-UPM, Madrid, Spain

**Keywords:** sFES, Muscle selectivity, Muscle fatigue, Multi-pad electrodes, Neuroprosthesis

## Abstract

Non-invasive neuroprosthetic (NP) technologies for movement compensation and rehabilitation remain with challenges for their clinical application. Two of those major challenges are selective activation of muscles and fatigue management. This review discusses how electrode arrays improve the efficiency and selectivity of functional electrical stimulation (FES) applied via transcutaneous electrodes. In this paper we review the principles and achievements during the last decade on techniques for artificial motor unit recruitment to improve the selective activation of muscles. We review the key factors affecting the outcome of muscle force production via multi-pad transcutaneous electrical stimulation and discuss how stimulation parameters can be set to optimize external activation of body segments. A detailed review of existing electrode array systems proposed by different research teams is also provided. Furthermore, a review of the targeted applications of existing electrode arrays for control of upper and lower limb NPs is provided. Eventually, last section demonstrates the potential of electrode arrays to overcome the major challenges of NPs for compensation and rehabilitation of patient-specific impairments.

## Background

A new generation of orthotic and prosthetic devices has started to include active elements capable of providing (or removing) energy to compensate and enhance human function. In this regard, the application of human muscles as actuators of orthotic systems by surface Functional Electrical Stimulation (sFES) is a promising technology [1]. FES systems were introduced as a method to externally activate the sensory-motor system in case of central nervous system (CNS) lesion [2, 3]. FES systems can be applied as motor neuroprostheses of motor functions for recovery in stroke patients [4, 5] or as means for compensation in assistive technologies, for example for control of walking and grasping after spinal cord injury (SCI) [6] or tremor suppression [7]. In general, available sFES systems for motor neuroprostheses face two major limitations, in addition to skin irritation and pain: a) insufficient selective activation of muscles and b) muscle fatigue as a reaction to muscle stimulation [8]. These two challenges remain open and the goal of this review is to assess the recent progress of research groups to overcome these limitations.

Consequently, the following question arises: How can selective and less fatiguing muscle activation be achieved with surface electrode arrays The review of the literature in this article is aimed to answer this question, providing a detailed revision of the state-of-the-art on selective sFES technology, their benefits, advantages and challenges. This work aims to revise both the available surface electrode arrays and the applied control strategies on this kind of sFES applications. The structure of this article is the following. Functional electrical stimulation and selectivity section provides an overview of the theoretical basis of sFES activation; feasibility of selective muscle activation through sFES is discussed. In Electrodes for muscle activation selectivity section novel solutions to design surface electrodes to improve muscle selectivity are revised. Selective control of muscle activation section presents a list of candidate applications of surface array electrodes in motor rehabilitation while Discussion section discusses in detail the current technology applied in most relevant available systems that address selective activation of muscles. Finally, conclusions are presented in Conclusions section.

### Methods

The studies included in this review are the result of a search in electronic literature and international congress proceedings. Electronic libraries such as SCOPUS, ScienceDirect, PubMed and IEEE Xplore were used. Proceedings from international congress EMBS, IFESS, NER, ICNR and ICORR were also included. The search criteria were studies that presented in decade 2003 to 2014. The keywords were “muscle selectivity”, “electrode array”, “sFES”, “multi-pad electrodes”, “muscle fatigue”, the inclusion criteria were:

- Studies presented a new electrode array system
- Use of electrode arrays or small electrodes grouped as an array in order to study muscle selectivity or fatigue.
- Stimulation strategy in muscle fatigue studies had to be asynchronous activation of each element of the array.
- FES had to be superficial and not implanted
- sFES application could be on forearm muscles or lower limb knee or plantar flexors/extensors

## Review

### Functional electrical stimulation and selectivity

Conventional surface electrodes that are individually applied to muscles are more suitable for stimulation of relatively large muscles that are close to the skin. However, such individual electrodes are limited to deliver stimulation to deeper muscles and to achieve fine control of groups of muscles [3]. As a result, selectivity needs to be significantly improved for applications of sFES to control the movements that involve multiple muscles or muscles that not innervate close to the skin. A classical example is the control of forearm movement, in which muscles are activated to generate forces at fingers and wrist joint [9]. In this context, several factors influence the quality of the movement induced by sFES. The movement generated by sFES with traditional electrodes depends on the position and the size of the cathode electrode [10], mainly due to the “overflow” phenomenon. The “overflow” phenomenon is described as the excitation of muscles that are adjacent to the target muscle, which results in undesired elicited movements. Additionally, size and type of the sFES electrode influence the pain threshold as well as the motor threshold of the stimulation [10, 11]. Current approaches to minimize the overflow phenomenon are based on invasive electrodes placed over (epimysial), around (cuff) or inside (intramuscular) the target muscle [8]. However, this type of electrodes requires a surgical procedure to be inserted into the target muscles. There are also alternative solutions based on percutaneous electrodes, which are placed in the target muscle without surgical procedures, although the risk of an infection is still high [8]. In summary, due to their invasive nature and possible medical complications, clinicians and patients are prone to avoid such type of electrodes. In contrast to this solution, transcutaneous electrodes are characterized by fast, non-invasive and simple use, which increases their acceptability by end-users.

Muscle activation selectivity can be achieved with conventional stimulation electrodes as long as the electric field generated under the electrode is adequately controlled. The modulation of the resulting electric field generated under the surface electrode that is aimed to reach the motor nerve and muscle fibers is a difficult task [12]. Current solutions control the stimulation parameters of the stimulator to achieve a moderation of the electric field generated under the surface of the electrode.

Another important challenge for the translation of sFES-based solution for clinical use is muscle fatigue. Muscle fatigue is defined as the progressive loss of capability of the stimulated muscle to be contracted [1]. In general, it can be said that muscle fatigue during artificial activation by sFES is developed faster if compared to physiological muscle activation. This is due to the fact that sFES technologies are not selective on muscle fibers recruitment since the stimulation applied at the skin surface recruits basically the same set of muscle fibers beneath a stable surface electrode, which is not the physiological approach for muscle contraction. Furthermore, although the negative effect of muscle fatigue induced by sFES is temporary, it is the main issue to achieve functional compensation or substitution of activities were muscle activation should be guaranteed for safety, such as standing or walking [3].

Over the last years, several studies have evaluated techniques for the mitigation of muscle fatigue during electrical stimulation. Muscle fatigue strategies can be separated in two main approaches: Closed-loop control strategies to control electrical stimulator parameters and advances in sFES electrodes’ technology. Each category separated has demonstrated that muscle fatigue appearance can be delayed. However, we believe that the combination of the two categories could improve the outcomes of each one stand-alone. A number of control techniques have been proposed in the literature as approaches managing the appearance of muscle fatigue in surface stimulation. Among others, PID control [13], adaptive control [14], fuzzy logic [15], neural networks [16] and adaptive sliding mode control [17], have been reported as alternatives to delay muscle fatigue. For detailed reviews on the literature on such control techniques the reader is referred to [18, 19] and [20]. These control techniques aim to modulate muscle fiber temporal recruitment either by varying pulse temporal characteristics (such as inter-pulse intervals or train frequency) or by predictive models that account for fatigue to control the stimulation [21–27]. Other studies testing invasive approaches to address sFES-induced fatigue have shown that cuff electrodes also contribute to delay the appearance of muscle fatigue. The former result is explained by a high degree of spatial selectivity when directly and selectively stimulating nerve fibers, which in turn allows choosing when muscle fibers are recruited depending on the tripole that is activated [28]. However, the practical drawbacks of using cuff electrodes have been exposed previously [8].

Besides the difficulty of controlling the electric field generated beneath the electrodes, there are differences in muscle characteristics and fatigue properties after SCI. As a result of disruption in nerve activation, loss of muscle mass and transformation of muscle fiber to type II fast-twitch fibers is observed [29]. In addition, other three major differences between CNS and sFES motor unit recruitment have an influence on muscle fatigue [3]. The first difference is how motor units are recruited, in synchronous or asynchronous mode [30]. The second difference is the order of recruitment of the different muscle fibers types [31, 32]. More information about muscle fibers’ types and their response can be found in the literature [33–37]. The third difference is that CNS fires action potentials that activate muscle fibers at a low frequency of 6-8Hz [36]. In contrast, sFES requires frequencies of titanic contraction (20Hz) or higher in order to avoid tremulous muscle contractions [36].

Recently, the scope of potential clinical applications of sFES has widened and with it, the required level of muscle activation selectivity and fatigue resistance. The former motivations and latest technology advantages have led to the development of new sFES systems that should ultimately achieve a variety of selective movements and delay muscle fatigue, by means of non-invasive surface electrode arrays.

### Electrodes for muscle activation selectivity

Advances in electronics and electrode design in the last decade, have led to a number of sFES systems that are able to implement theoretical muscle contraction principles in sFES practice. These systems apply an electric field with irregular shape and size with the use of electrode arrays [38]. An electrode array consists of a set of small electrodes (also known as pads) arranged in an array [39]. These systems were firstly proposed as a solution to electrode misplacement over the muscle since these allow for the relocation of the stimulated area with a fixed physical electrode location [40]. Another advantage of the electrode array is the possibility to place both anode and cathode electrodes over a single array. In this way, the delivery of stimulation can be simplified since only one element (array) has to be placed over the human limb and less time is required to find the correct position of the electrode [40]. Each pad in one electrode array can be activated independently to control the spatial and temporal distribution of electrical current field [40] and hence the excitation of different motor units. In that way, the pads that are better located over the target muscle can be selectively activated to generate a specific target movement. The first study found in literature of this kind of electrodes was developed by Nathan R. in 1979 [41], he proposed the electrode belts that consisted of a row of electrodes that was positioned perpendicular to the forearm in order to achieve individual activation of forearm muscles. The work of Lawrence et al. [42], Kuhn. [39] and Keller et al. [40] marked a line for the direction of the following studies on muscle selectivity. They were the first to develop electrode arrays with the structure presented in this work and they study the design of the array, the current distributions under it by developing advanced wrist control systems.

To the best of our knowledge, eight different array electrodes have been presented in the literature until the date. We categorized the electrode arrays that we found in the literature as follows:

1. Plastic flexible substrate electrodes: Actitrode [12, 43], INTFES [44], Chen [45], HYPER, MUNDUS [46], made by flexible printed circuit board on a polycarbonate.
2. Other textile electrodes: Smart Electrode [42, 47] and Smartex [48], made by silver coated fibers embroidered into square elements arranged in an array.

A third innovative technique to manufacture electrode arrays was presented by Yang et al. [49]. A screen-printed flexible and breathable fabric electrode array (FEA) fabricated entirely by screen printing the active electrode array directly onto a standard fabric was employed in this design. The electrode array consisted of four printed functional layers. The solution is proposed to reduce the costs associated with the embroidery technique [50] and the conductive path constraints of weaving and knitting approaches [48, 51].

**Fig. 1:**
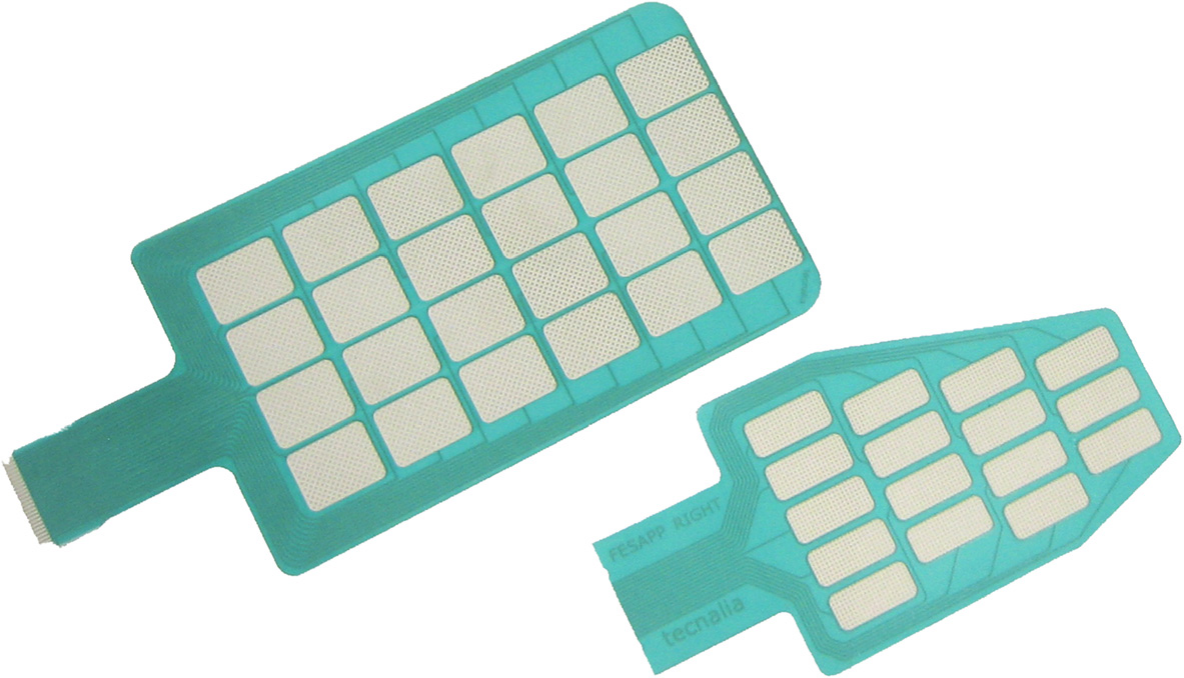
Two examples of INTFES electrodes for upper limb sFES applications

Table 1 contains details of material, electrode pads and sizes from above mentioned electrode arrays. The electrode pads were round shaped in Smart, Actitrode, Smartex, and Chen electrode arrays, while rectangular shape was preferred in INTFES (see Fig. 1), HYPER (see Figs. 3 and 4), Yang and MUNDUS electrode arrays.

**Table 1:** Comparison of main features of electrode arrays found in literature

Electrode array	Material	Number of pads	Array’s structure	Pad’s shape	Pad’s size	Gap's size	Electrode’s dimensions
Smart Electrode	textile silver coated fibres	16-pads	4 × 4	round	10 mm × 10 mm	2 mm	5 cm × 5 cm
		64-pads	8 × 8				10 cm × 10 cm
Actitrode	plastic flexible substrate	24-pads	6 × 4	round	1 cm	0.9 cm	8 cm × 5 cm
		12-pads	6 × 2		1.2 cm		
INTFES (by Tecnalia)	plastic flexible substrate	16-pads	4 × 4	oval, rectangular		2 mm	
Smartex (by Smartex)	textile	25-pads	5 × 5	round	1 cm	5 mm	9 cm × 9 cm
Chen	plastic flexible substrate	30-pads	6 rows of 5 pads	round	1 cm	3 mm	8.5 cm × 7.2 cm
HYPER (by Tecnalia)	plastic flexible substrate	16-pads	4 × 4	rectangular	2.6 cm × 0.6 cm	2 mm	
FEA	screen-printed flexible and breathable fabric	24-pads	4 × 6	oval	0.75 cm × 1.25 cm	0.75 cm	11.5 cm × 5.5 cm
MUNDUS (by Tecnalia)	textile + plastic flexible substrate	78-pads	6 separated arrays	rectangular		2 mm

### Selective control of muscle activation

Two main types of applications of surface electrode arrays for muscle stimulation have been found in the literature: i) selective control of forearm muscles through modulation of the electric field under the surface electrode and ii) management of muscle fatigue in leg muscles, through asynchronous activation of motor units. In both scenarios, the key challenge is to apply electrode arrays to selectively activate different motor units with individual pads or combinations of pads.

#### Upper limb selective sFES applications

Most of the studies on control of upper limb movement were focused on developing algorithms for selecting the best-located pads. Popovic et al. [52] underlined the importance of muscle fatigue in upper limb function tasks. Table 2 contains details of sFES systems with electrode arrays in the upper limb. The following section revises the hardware platforms that have been proposed to analyze the response of sFES and addressing the selection of best-located pads over targeted muscles.

**Table 2:** Comparison of main selectivity study platforms found in literature

**Platform**	Lawrence et al., 2006 [54]	O'Dwyer et al., 2006 [56]	Popovic et al, 2009 [57]	Malesevic et. al, 2010 [58]	Malesevic et al, 2012 [59]	Schill et al, 2009 [60]	Chen et al, 2007 [45]	Koutsou et al, 2013 [61]	Exell et al, 2013 [62]
**Array Electrode**	Smart Electrode 64-pads	2 x 2 round	Actitrode, 24-pads, round	INTFES, 16-pads, oval	INTFES, 16-pads, oval	2x3 round	own electrode array	Smartex electrode array	FEA, 4x6 round
**Electrical stimulator**	Compex Motion2. 4-channel biphasic asymmetric	Neurotech NT2000, 6-channel biphasic asymmetric	UNAFET, 4-channel biphasic asymmetric	INTEFES, 1-channel with a demultiplexer for 32-pad electrodes monophasic rectangular	INTEFES, 1-channel with a demultiplexer for 32-pad electrodes monophasic rectangular	MotionStim 8, 8-channel biphasic	NM III	INTEFES, 1-channel with a demultiplexer for 32-pad electrodes monophasic rectangular	Modified Odstock stimulator
**Upper Limb functions**	fingers' flexion	Wrist flexion-extension,abduction-aduction, Fingers's flexion	Forearm pronation-supination, wrist flexion-extension, abduction-adduction, fingers's flexion-extension	wrist flexion-extension, fingers' flexion-extension	wrist flexion-extension, fingers' flexion	wrist flexion-extension, abduction-adduction	Wrist joint, fingers' felxion-extension	Forearm pronation-supination, wrist flexion-extension, abduction-adduction, fingers's flexion-extension	Wrist joint, fingers' felxion-extension
**Sensors**	miniature load cells	bend sensors, accelelometers	goniometers	goniometers, accelelometers	bend sensors	bend sensors	CyberGlove	bend sensors, accelelometers	Data glove, twin-axis electrogoniometer
**Stimulation Strategy**	Sequentially activation of pads region during 5 s	Sequentially activation of a different combination of pair electrodes during 3 s	Sequentially activation of pads during 4 s	Single current pulses via each pad, with frequency of 2 Hz.	Sequentially activation of pads during 2 s	Single current pulses via each pad	Sequentially activation of pads	Sequentially activation of pads during 2 s	Sequentially activation of pads region(blocks)
**Pad Selection**	Automatic, regions with higher forces	Automatic, comparison with target movement	Automatic, cost function	Automatic, ANN	Automatic, cost function	semi-automatic, cost function	non-automatic, pas with higher amount of movement	Automatic, cost function	Automatic, ILC
**Portability**	NOT Portable	Portable	portable	portable	portable	portable	portable	portable	portable

##### Hardware classification

Different types of sensors can be applied to determine the response to stimulation. We categorized the platforms found in the literature as follows: 1) force/torque generation [53–55] and 2) kinematic generation [45, 56–61]. Kinematic sensors are becoming preferred for these purposes because of portability and simple use. Accelerometers were widely preferred in seminal works [56–58, 61], although later low-cost flex sensors were considered as an alternative [59, 60, 62].

##### Algorithm classification

Different concepts have been proposed to develop algorithms for the selection of the best-located pads. We found studies that analized the force and determined if the activated pad contracts the desired muscle [54, 55]. Other type of systems compared the generated movement with respect to a predefined movement [56]. Also, computational algorithms have been proposed to compare the generated movement with the desired movement and use this factor to qualify the activated pad [57, 59–61]. Artificial Neural Networks (ANN) and Iterative Learning Control (ILC) were also proposed for the selection of the pads [58, 62].

#### Lower limb selective sFES applications

Five studies that have addressed muscle fatigue management with asynchronous selective activation of lower limb muscles were revised [63–67]. Main characteristics of these studies can be found in Table 3. We focus on two key characteristics for each study: force production assessment and stimulation strategy.

**Table 3:** Comparison of main features of lower limb fatigue resistant strategies

Fatigue strategy	Muscle fatigue definition	Fatigue metric	Muscle group	Stimulation strategies & electrodes	Subjects	Results
Popovic et al. 2009 [63]	70 % decrease of max torque	Fatigue Interval	Quadriceps	Synchronous single electrode vs Asynchronous 4 smaller electrodes	6 complete SCI patients	150 % increase of fatigue interval with electrode array
Malesevic et al. 2010 [58]	70 % decrease of max torque	Fatigue Interval	Quadriceps	Synchronous single electrode vs Asynchronous 4 smaller electrodes	6 complete SCI patients	Synchronous: 31 % increase of post-therapy muscle fatigue resistance.
				20 daily sessions		Asynchronous: 4 % increase of post-therapy muscle fatigue resistance.
Nguyen et al. 2011 [64]	Torque decrease of 3 dB	Fatigue Index, Fatigue Time, Torque-Time-Interval	Tricep Surae	Synchronous single electrode vs Asynchronous 4 smaller electrodes	1 complete SCI	Asynchronous stimulation: higher torque values for a longer period of time
Sayenko et al. 2013 [67]	Torque decrease of 3 dB	Fatigue Index	Knee flexors/extensors, plantar flexor/dorsiflexor	Synchronous single electrode vs Asynchronous 4 smaller electrodes	15 able-bodied subjects	Asynchronous stimulation higher fatigue resistant than synchronous
Sayenko et al. 2014 [65]	They studied muscle contraction properties	Torque-Rise Time, Rate of torque development, Half-Relaxation-Time, Rate of torque relaxation	Tricep Surae, right gastrocnemius	Synchronous single electrode vs Asynchronous 4 smaller electrodes	15 able-bodied subjects	Amplitude of M-waves depends on the location of the stimulated pad electrodes. Peaks on M-waves on ascending phase of synchronous stimulation are fused as fatigue occurs.

##### Assessment of force production

Muscle fatigue can be defined in different ways. The approach adopted in Popovic et al. [63] and Malesevic et al. [66] was defined as the decrease to 70 % of the maximum of the knee torque, while in Nguyen et al. [64] and Sayenko et al. [67] was defined as the decrease of 3db of the maximum torque. Furthermore, Sayenko et al. [65] used a group of muscle properties to study muscle fatigue as well. Different metrics have been used to assess force production: time interval before muscle fatigue appears [63, 66], muscle fatigue index (torque at the end of stimulation), fatigue time (time passed by until torque decreases 3 dB), torque-time interval (integral of torque during stimulation time) [64, 67]. Sayenko et al. [65] used a protocol that included the following measures to describe muscle contraction and relaxation: 1) torque rise time in ascending phase, 2) rate of torque development in ascending phase, 3) half-relaxation time in descending phase and 4) rate of torque relaxation.

##### Stimulation strategies

Different stimulation strategies have been proposed as a way to mimic CNS and activate asynchronously different muscles fibers and muscles. The stimulation strategies for the lower limb share the following characteristics: a) asynchronous stimulation achieved with the use of four electrodes, and b) stimulation frequency of single electrode stimulation that is close to the sum of individual stimulation frequencies of all pad electrodes. Stimulation frequencies in single electrode strategy were similar 40Hz [63–65, 67] and 30Hz [66]. In asynchronous electrode pad strategy, each pad is activated sequentially with a delay from the previous one. Stimulation frequency of muscle group is reduced to 16Hz [63, 66] and 10Hz [64, 65, 67], while electrode pad stimulation frequency is the same as in single electrode strategy.

### Discussion

Two main materials had been used in electrode array fabrication: textile and plastic flexible substrate. Hydrogel membrane in plastic electrode arrays offers a better contact with the electrode substrate. Regardless of the electrode’s material, a layer of a hydrogel membrane between the electrode and the skin is needed in order to avoid skin irritations and pain [12, 44–48]. Furthermore, the impedance of the used hydrogel membrane in combination with pad’s and gap’s size influence on muscle activation selectivity [68]. However, the electrode array proposed by Yang et al. [49] reported no need of use of hydrogel membrane and resulted in higher repeatability of movement than the INTFES [44].

**Fig. 2:**
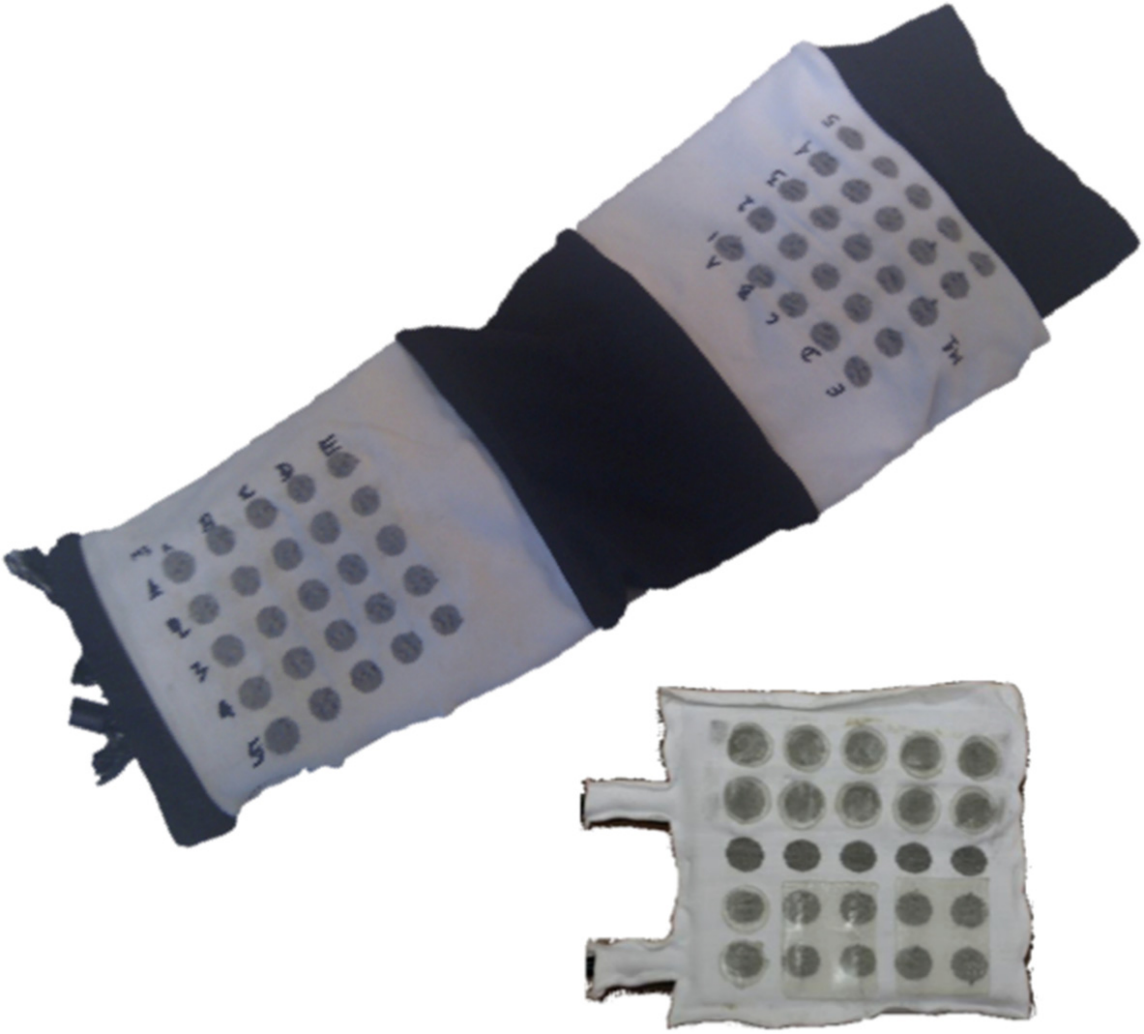
Two versions of SMARTEX electrode. Single electrode array version right corner and 4-electrode array garment for full upper limb sFES applications

**Fig. 3:**
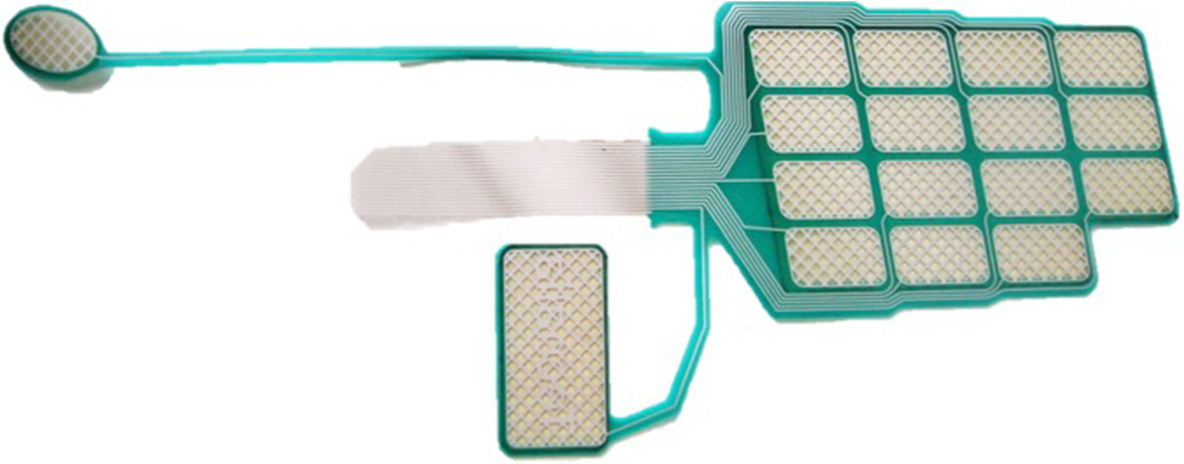
HYPER electrode for right wrist extensors surface stimulation. Electrode for wrists flexors is symmetrical to this one

**Fig. 4:**
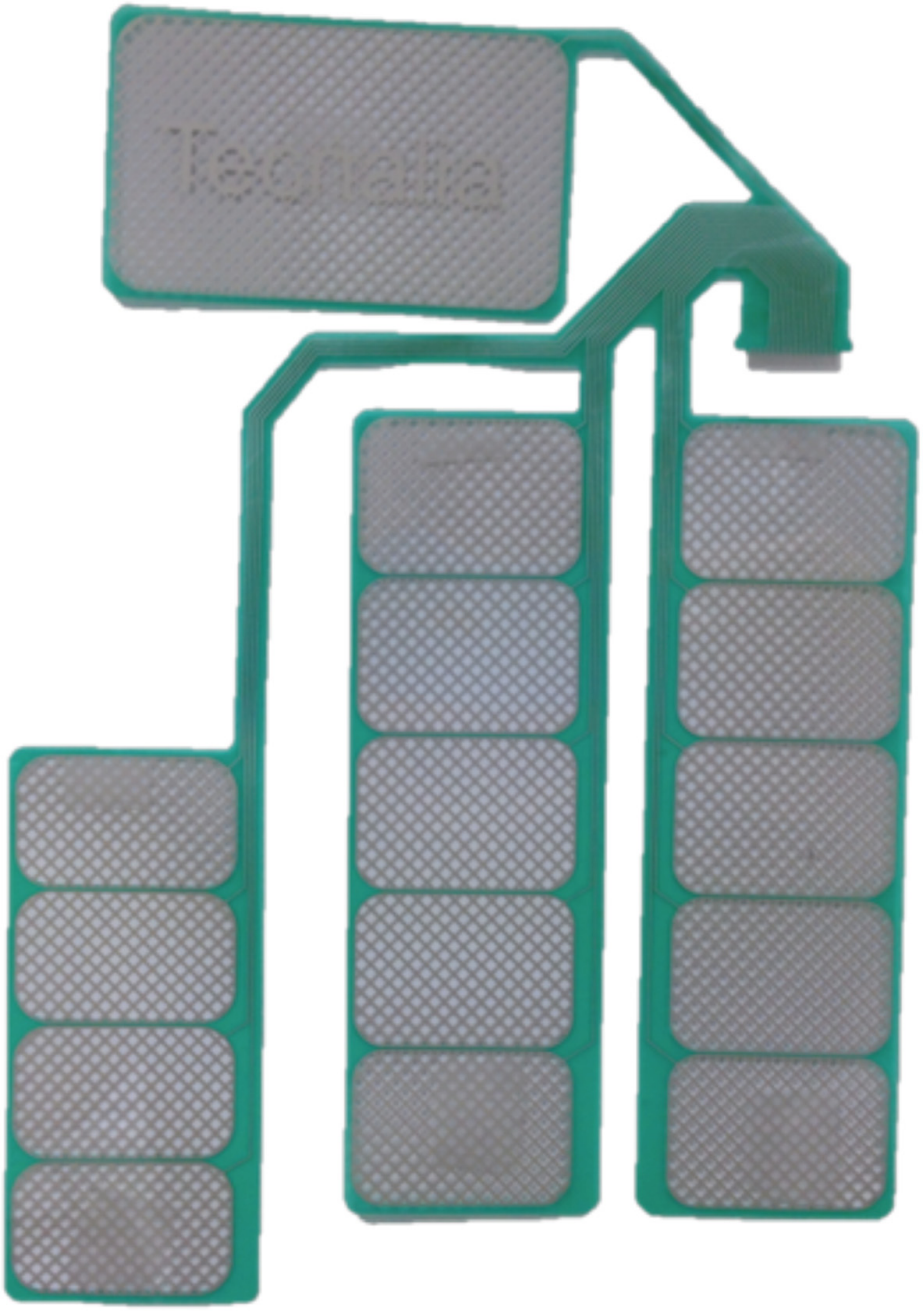
HYPER electrode for lower limb quadriceps surface stimulation

Two of the most important characteristics of an array electrode are the size of the forearm and the design of the array [59]: the size of the array electrode should be scaled according to the size of the forearm of the patient. The design of the array structure should take into account the shape, size and position of the target muscle group in order to be able to cover all its superficial area. Kuhn et al. [39] were the first to mark the importance of the array design on muscle selectivity. The authors developed a Finite Element and a nerve model in order to find the gel resistivities and gap sizes more adequate for muscle selectivity in array electrodes. The simulation results indicated that a high resistance gel reduces the activated area under the gaps but increases muscle selectivity. Pad electrode dimension influences muscle selectivity depending on the size of the target muscle. In addition, it has been proven that the size of the electrode pad over a single muscle does not change the number of activated muscle fibers [69], but it is demonstrated that is correlated to skin irritations and pain. Control of upper limb muscles with sFES electrode arrays can be achieved with relatively low intensity (50 mA). On the other hand, artificial control of the lower limb muscles with surface electrodes demands higher range (100 mA) and thus, pad electrode design should address avoidance of skin irritations, pain and burns. Some electrode arrays were designed specifically for upper limb applications, such as in Smartex (see Fig. 2) and MUNDUS. Smartex was an upper limb garment that integrates four 25-pads electrodes arrays for EMG recordings and FES stimulation of main muscle groups of the upper limb (biceps, triceps, wrist flexors and extensors). MUNDUS consisted of six bendable embedded customizable stimulation arrays for stimulation of wrist movements. HYPER electrodes were designed and developed as a complete solution for the whole body: upper limb (see Fig. 3), lower limb (see Fig. 4), shoulder, back, and gluteus. Each electrode array was adapted to each muscle group. For example, for wrist functions, the design was separated into: a) first part consisted of 15 pads to stimulate finger flexors or extensors and forearm pronator or supinator, and b) second part consisted of 1 pad to stimulate thumb flexors or extensors, see Fig. 3. Popovic-Maneski et al. [70] presented a very detailed work on the design of another version of the INTFES electrode for forearm sFES applications. They underline the variety of the stimulation sites between patients that resulted in designing a rather large electrode array.

The main difference between electrode arrays [12, 44, 46–49] and individual electrodes is that each pad can be independently activated and therefore can be converted in an electrode of an irregular shape. Another case is, for example, the proposed by Chen et al. [45] in which an electrode array of 30-pads was proposed that could be activated in groups of five pads on the same line. This resulted in possible active electrodes of rectangular shape but with different size.

Electrode arrays to be applied at upper limb NPs, require a custom configuration method and hardware. The stimulation strategy chosen for the configuration method drastically affect the time duration of this phase. In this regard, sequential activation of pads (or a group of pads) for a short period of seconds was the most used strategy [45, 54, 56, 57, 59, 61, 62]. However, Malesevic et al. [58] and Schill et al. [60] proposed a faster method: a single pulse stimulation strategy for each pad. Furthermore, RT-time sFES control systems need the best-located pads configuration method to be fully automatic [54, 56–59, 61, 62] in order to be able to adjust the stimulation pattern as the forearm position changes. Alternatively, other works such as Schill et al. [60] and Chen et al. [45] do proposed a different method applying semi- and non-automatic configuration methods, respectively.

Lower-limb NPs do not require complex electrode array configurations. The revised lower limb NPs with multi-pad electrodes are focused on strategies for asynchronous independent activation of pad electrodes. A common finding was that asynchronous stimulation of the pads of electrode arrays delays the appearance of muscle fatigue [63–67]. It has been demonstrated that asynchronous stimulation with electrode arrays can increase the time interval to generate fatigue up to 150 % in comparison with single electrode configurations [63]. A recent study that tested this technique to promote lower limb muscle function in SCI patients confirmed this finding [66]. However, the former study concluded that electrode array should not be used in applications to train for muscle fatigue resistance.

Interestingly, an investigation [65] of asynchronous stimulation with electrode arrays on the gastrocnemius muscles in able-bodied subjects demonstrated that the amplitude of the M-waves at each muscle portion is dependent on the location of the stimulation electrode pad. This led to suggest that different sets of muscles fibers are excited each time a different electrode pad is activated [65]. Overall results in those studies were independent of pad electrode or muscle size. Furthermore, Malesevic et al. [59] demonstrated that asynchronous activation with different FES parameters could be applied for grasping. Interestingly. Furthermore, it has been demonstrated that synchronous activation with electrode arrays may increase muscle fatigue in comparison with a single electrode of the same size due to muscle fibers that fall under the region of the gaps between pads [69]. Lastly, asynchronous stimulation needs independently activated pads and thus, the number of pad arrays that can be activated with different FES parameters should be maximized in future designs.

#### The future of multi-pad electrode based FES

Besides the above revised considerations, electronic design and its effects on the ability to dynamically define the stimulation parameters (e.g., pulse amplitude, shape, width, repetition frequency, train duration), are crucial to achieve efficient selective muscle activation with electrode arrays. Latest studies and advances in sFES highlight the need of flexible waveforms generators and multi-channel systems with real-time connectivity and with an independent configuration of stimulation parameters per channel. Electrode arrays can contain dozens of electrodes that have to be connected to the stimulator and moreover stable current stimulator is not trivial to design using compact electronic components. There are research prototypes attempting to solve this matter by means of custom microelectronic design. However, power management and dissipation are major concerns and still open issues in these type of prototypes [71]. Stimulators of these characteristics are at the time only available for research purposes [44, 71].

At the moment, there are no studies that include platforms of electrode arrays that account for real-time FES control and electrode array reconfiguration. In the future, the combination of closed-loop control strategies with best-located pad electrodes algorithms should be the subject of applications to control the upper limb muscles, while in the lower limb closed-loop fatigue control with low-frequency stimulation should be developed.

Finally, the studies included in this review were conducted with limited number of subjects and short intervention periods; therefore, clinical studies with larger numbers of patients and rehabilitative approaches should be conducted.

## Conclusions

Muscle activation selectivity and fatigue are the main challenges in sFES. Over the last decade several investigations have attempted to artificially induce motor unit recruitment in order to achieve high level of muscle selectivity and improved fatigue response. Research studies on upper limb NPs with electrode arrays have led to significant improvement of muscle activation selectivity. However, further studies on closed-loop control selectivity activation strategies need to be developed, clinical studies with impaired subjects are necessary in order to certify the obtained results until now and new microelectronics system stimulators are needed to be available for clinical use.

## Abbreviations

ANN, Artificial Neural Networks; CNS, Central Nervous System; EMG, Electromyography; FEA, Fabric Electrode Array; FG, Fast, Glycolytic; ILC, Iterative Learning Control; NPs, Neuro-Prostheses; PID, Proportional–Integral–Derivative; SCI, Spinal Cord Injury; sFES, Surface Functional Electrical Stimulation; SO, Slow, Oxidative.
